# Synergistic Effects of PPAR*γ* Ligands and Retinoids in Cancer Treatment

**DOI:** 10.1155/2008/181047

**Published:** 2008-05-28

**Authors:** Masahito Shimizu, Hisataka Moriwaki

**Affiliations:** Department of Medicine, Gifu University Graduate School of Medicine, Gifu 501-1194, Japan

## Abstract

Peroxisome proliferator-activated receptors (PPARs) are members of the nuclear receptor superfamily. The activation of PPARs by their specific ligands is regarded as one of the promising strategies to inhibit cancer cell growth. However, recent clinical trials targeting several common cancers showed no beneficial effect when PPAR ligands are used as a monotherapy. Retinoid X receptors (RXRs), which play a critical role in normal cell proliferation as a master regulator for nuclear receptors, preferentially form heterodimers with PPARs. A malfunction of RXR*α* due to phosphorylation by the Ras/MAPK signaling pathway is associated with the development of certain types of human malignancies. The activation of PPAR*γ*/RXR heterodimer by their respective ligands synergistically inhibits cell growth, while inducing apoptosis in human colon cancer cells when the phosphorylation of RXR*α* was inhibited. We herein review the synergistic antitumor effects produced by the combination of the PPAR, especially PPAR*γ*, ligands plus other agents, especially retinoids, in a variety of human cancers. We also focus on the phosphorylation of RXR*α* because the inhibition of RXR*α* phosphorylation and the restoration of its physiological function may activate PPAR/RXR heterodimer and, therefore, be a potentially effective and critical strategy for the inhibition of cancer cell growth.

## 1. INTRODUCTION

Peroxisome proliferator-activated receptors (PPARs) are members of a superfamily of nuclear hormone receptors comprising three isoforms, PPAR*α*, PPAR*β*/*δ*, and PPAR*γ*, which act as ligand-activated transcription factors. PPARs play key roles in energy homeostasis by modulating glucose and lipid metabolism and transport. Through these metabolic actions, PPARs can regulate cell proliferation, differentiation and survival [1, 2]. PPARs also control immune and inflammatory responses [3]. Because these physiological activities of PPARs are closely associated with normal cell homeostasis, the aberrant expression and function of PPARs have been observed in a variety of human malignancies. Moreover, these reports also suggest the possibility that targeting PPARs might be a critical strategy for inhibiting the development and growth of cancers. Indeed, numerous in vivo and in vitro studies have demonstrated that PPAR agonists, especially, PPAR*γ* ligands can inhibit cell growth, cause apoptosis, and thus exert antitumor effects in various types of human malignancies [4–6]. Based on the antigrowth and prodifferentiation action of PPARs, several clinical studies have been conducted using the PPAR ligands in human cancers. However, with the exception of a small trial on liposarcomas, the clinical trials have so far indicated that the PPAR agonists may not be useful as a monotherapy for advanced malignancies [7–10].

On the other hand, recent preclinical studies show absorbing evidence that the combined treatment with PPAR ligands plus a variety of other agents can cause a synergistic effect to inhibit growth in cancer cells. For instance, we recently found that the activation of PPAR*γ*/RXR heterodimer by their respective ligands synergistically inhibited cell growth and induced apoptosis in human colon cancer cells [11]. Therefore, the aim of this paper is to review the possibility that the combined usage of the PPAR ligands with other agents may therefore be a critical strategy for the treatment of certain types of human cancers. We also review the significance of the aberrant phosphorylation of retinoid X receptor (RXR), which is a heterodimeric partner for PPARs, as described in the next section.

## 2. RXRs AND PPARs

**Figure 1:**
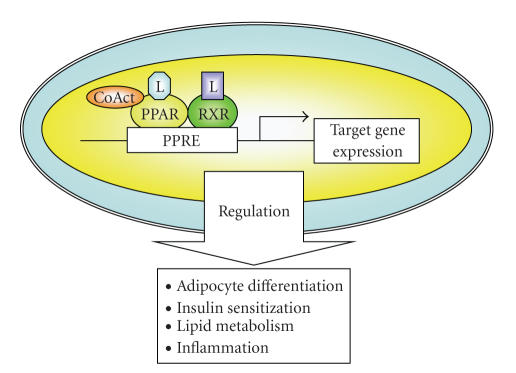
PPAR activation pathway and transcriptional regulation of target genes. After ligand binding, PPARs form heterodimers with RXR in the nucleus. The PPAR/RXR heterodimers interact with transcriptional coactivators (CoActs) and bind to sequence specific PPRE located in the target genes that control glucose and insulin homeostasis, lipid metabolism, inflammation, and cellular differentiation. L: ligand.

RXRs and retinoic acid receptors (RARs), both of which are composed of three subtypes (*α*, *β*, and *γ*), are also members of the nuclear hormone receptor superfamily. The ligands for RXRs and RARs are the retinoids, a group of structural and functional analogues of vitamin A, and the retinoids have a profound effect on such cellular activities as growth, differentiation, apoptosis, and morphogenesis primarily through binding to RXRs and/or RARs. A small portion of dietary retinoids is converted to retinoic acid (RA), which is an active metabolite of the retinoids. RXR is specific for the 9-cis RA, while RAR binds both 9-cis RA and all-trans RA (ATRA). The nuclear retinoid receptors are ligand-dependent transcription factors that bind to the retinoic acid receptor responsive element (RARE) and retinoid X receptor responsive element (RXRE), which are present in the promoter regions of retinoid responsive target genes, thereby modulating the gene expression [12, 13]. Other nuclear receptors, including PPARs, also require RXR as a heterodimeric partner in order to exert their function. After ligand binding, PPARs can regulate target gene expression by binding to the peroxisome proliferator responsive element (PPRE) in target genes as a heterodimer with RXRs (see Figure 1) [14, 15]. Therefore, RXRs play a fundamental role in controlling normal cell proliferation and metabolism and act as a master regulator of nuclear receptors. Among the retinoid receptors, RXR*α* is thought to be one of the most important receptors with respect to the regulation of the essential effects of cell activities.

## 3. STRUCTURE OF RXR*α* AND SIGNIFICANCE OF RXR*α* PHOSPHORYLATION

RXRs have a variable N-terminal domain (A/B domain; AF-1), a highly conserved DNA-binding domain (DBD), a nonconserved hinge, and a moderately conserved C-terminus including the ligand-binding domain (LBD). *Transcriptional activation is mediated by LBD, which contains four more-or-less overlapping surfaces: a ligand-binding pocket for the binding of small, lipophilic molecules, a transactivation domain (AF-2 or helix 12), a cofactor binding surface, and a dimerization surface [16].* ** Recent studies revealed that phosphorylation processes are critical for the transcriptional activity of RAR/RXR heterodimers. Bruck et al. [17] reported that the activation of c-Jun N-terminal kinases (JNKs) induces phosphorylation of both at three residues (serine 61, serine 75, and threonine 87) located in the N-terminal AF-1 domain and one residue (serine 265) in the Omega loop in LBD (AF-2 domain) of RXR*α*. The RA-induced phosphorylation of the same three residues in the AF-1 domain is required for the cooperation of RXR*α* with RAR*γ* for maximal transcriptional activity [18]. The phosphorylation of RXR*α* in its N-terminal domain plays a role to activate a subset of RA-responsive genes and for the antiproliferative effect of RA [19]. These findings suggest that RXR*α* “positively” regulates the transactivation of target genes through phosphorylation [20].

On the other hand, there are some contrary reports which show the phosphorylation of RXR*α* to “negatively” modulate the function of its heterodimeric binding partners. Indeed, MAPK-mediated phosphorylation of the RXR*α* LBD impairs the transcriptional activity of RXR/RAR [21, 22] and RXR/vitamin D_3_ receptor (VDR) [23]. These “negative” effects of RXR*α* via its phosphorylation might be associated with certain types of human diseases, including cancer [20]. In the next section, we review the specific roles of the aberrant phosphorylation of RXR*α* in carcinogenesis, especially focusing on the development of hepatocellular carcinoma (HCC).

## 4. RXR*α* PHOSPHORYLATION AND CANCER

**Figure 2:**
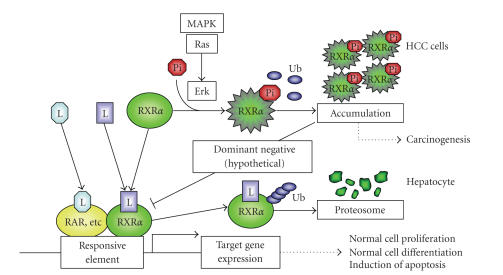
A schematic representation of RXR*α* phosphorylation in HCC cells. In normal hepatocytes, when the ligand (retinoid) binds to and activates RXR*α*, the receptor becomes able to heterodimerize with other nuclear receptors, including RAR, and then activates the expression of the target genes, which may regulate normal cell proliferation and differentiation, by binding to the specific responsive element. In HCC cells, the Ras/MAPK pathway is highly activated and phosphorylates RXR*α* at serine residues, thus impairing the functions of the receptor. Therefore, the accumulated p-RXR*α* interferes with the remaining normal RXR*α*, presumably, in a dominant negative manner, thereby playing a critical role in the development of HCC. L: ligand. Ub: ubiquitin.

Abnormalities in the expression and function of retinoids and their receptors play an important role in influencing the development of various human malignancies and, therefore, might be critical targets for cancer chemoprevention and/or chemotherapy [24]. Specifically, we previously reported that hepatocarcinogenesis is accompanied by an accumulation of the phosphorylated (i.e., inactivated) form of RXR*α* and the inhibition of RXR*α* phosphorylation may thus be an effective strategy for preventing the development of HCC. Initially, we showed that the RXR*α* protein is anomalously phosphorylated at a specific site of the serine/threonine residues and is accumulated both in human HCC tissue as well as in HCC cell lines [22]. Phosphorylation at serine 260 of RXR*α*, a consensus site of mitogen-activated protein kinase (MAPK), is closely linked to its retarded degradation, low transcriptional activity, and the promotion of cancer cell growth, and the abrogation of phosphorylation by MAPK-specific inhibitors restores the degradation of RXR*α* in a ligand-dependent manner [22, 25]. In addition, the phosphorylated form of RXR*α* (p-RXR*α*) is also resistant to ubiquitination and subsequent proteasome-mediated breakdown in both human HCC tissues and a human HCC cell line, whereas RXR*α* protein is unphosphorylated and highly ubiquitinated in the normal liver and in nonproliferating hepatocyte cultures [26]. The phosphorylation of RXR*α* abolishes its ability to form heterodimers with RAR*β*, thus resulting in the loss of cell growth control, resistance to retinoids, and the acceleration of cancer development [27]. These findings suggest that the accumulation of p-RXR*α* (i.e., nonfunctional RXR*α*), which can escape from the proteasome-mediated degradation system, may interfere with the function of normal RXR*α* in a dominant-negative manner, thereby playing a critical role in the development of HCC (see Figure 2) [28].

In addition to HCC, a malfunction of RXR*α* due to a posttranslational modification by phosphorylation is also associated with the development of other types of human malignancies. We recently reported that RXR*α* protein is highly phosphorylated and also accumulated in human colon cancer tissue samples as well as human colon cancer cell lines, while the levels of expression of p-RXR*α* do not increase in normal colonic epithelial cells; RXR*α* protein is phosphorylated in 75% of colorectal cancer tissues when compared with corresponding normal colon epithelial tissues [11]. Similar results have also been observed in human pancreatic cancer (manuscript in preparation). Moreover, Kanemura et al. [29] reported the abnormal phosphorylation of RXR*α* protein to play a role in the enhancement of cell proliferation, while producing an antiapoptotic effect, and also presumably acquiring RA-resistance in HL-60R human leukemia cells. In addition to these malignancies, full-length RXR*α* is anomalously phosphorylated and accumulated in leiomyoma when compared to myometrial cells and this is associated with a resistance to ligand-mediated ubiquitination and a delay in the receptor proteolytic degradation [30].

What are the precise mechanisms by which phosphorylation of RXR*α* loses its transcriptional activity? Recent studies indicate that the phosphorylation of RXR*α* can regulate the function of its heterodimeric binding partners. For instance, Solomon et al. [23] reported that phosphorylation of RXR*α* at serine 260, which is located in the Omega loop of the LBD, results in the attenuation of ligand-dependent transactivation by RXR/VDR complex in human keratinocytes, thus resulting in the induction of malignant transformation. The residues located in the AF-2 domain are also phosphorylated in response to stress agents, including JNKs and MKK4/SEK1 [21, 31], among which serine 265 located in the Omega loop [31], and these phosphorylations are closely linked to inhibit the transcription of RA target genes. The phosphorylation of RXR*α* at serine 260 is also associated with retinoid resistance [22, 27]. Therefore, these findings indicate that RXR*α* phosphorylation, which occurs at specific residues located in the Omega loop of the LBD, is apparently associated with a malfunction in the retinoid-dependent signaling pathway. The Omega loop, located between helices H1 and H3, is a very flexible and dynamic region that moves substantially during the conformational rearrangement that accompanies ligand binding to the LBD [32]. It has therefore been proposed that phosphorylation of the residues in this loop might alter the dynamics of this region and create conformational changes within the LBD, thus disrupting the interactions with coactivators and therefore inhibiting the activation of RA-responsive genes [17, 33].

## 5. PHOSPHORYLATED RXR*α* IS A CRITICAL TARGET FOR CANCER TREATMENT

The above findings support the possibility that the inhibition of RXR*α* phosphorylation and the restoration of its physiological function as a master regulator of nuclear receptors must be an effective strategy for controlling cell growth in various types of human cancers. It has been shown that the new synthetic retinoid, acyclic retinoid (ACR, NIK-333: Kowa Pharmaceutical Company Ltd., Tokyo, Japan), which was originally developed as an agonist for both RXR and RAR [34, 35], can restore the function of RXR*α* by inactivating the Ras-Erk signaling system and thereby inhibiting RXR*α* phosphorylation [25]. Practically, this agent has demonstrated several beneficial effects in experimental studies both in vivo and in vitro. For instance, ACR inhibited chemically induced hepatocarcinogenesis in rats as well as spontaneously occurring hepatoma in mice [36]. This agent also inhibited growth and induced apoptosis in human HCCderived cells [37–42]. Similar growth inhibitory effects are also observed in other types of human cancer cells, such as squamous cell carcinoma or leukemia cells [43, 44].

In addition, we also confirmed the chemopreventive effect of ACR on recurrent and second primary HCCs in patients who received anticancer treatment for an initial HCC in a double-blind and placebocontrolled clinical study. Namely, the oral administration of ACR for 12 months significantly reducedthe incidence of posttherapeutic recurrence of HCC and improved the survival rate in patients who underwent potentially curative treatments, without causing any severe adverse effects [45–47]. These findings suggest that ACR is a promising agent for the chemoprevention of HCC and that p-RXR*α* may be a critical target for the chemoprevention and/or treatment of some types of human cancers, including HCC, which show the accumulation of p-RXR*α* protein.

## 6. SYNERGY BETWEEN PPAR*γ* LIGANDS AND RETINOIDS IN CANCER

Since RXR forms a permissive heterodimeric complex with PPAR, and the activation of PPAR*γ* exerts antigrowth effects in cancer cells [4–6], it seems to be reasonable that the combination of RXR and PPAR*γ* agonists may offer new therapeutic strategies for various types of human malignancies. Firstly, Tontonoz et al. [15] reported that the combined use of PPAR*γ* and RXR*α* specific ligands is able to trigger terminal differentiation of primary human liposarcoma cells in vitro. This result suggests that the combination of these ligands may be a useful therapy for the treatment of liposarcoma [15]. Beneficial effects for the combined treatment with PPAR ligands plus retinoids are extensively reported in preclinical studies of the hematologic malignancies [48–51]. Therefore, the combination of PPAR*γ* ligand with RXR agonist or RAR agonist can enhance the differentiating and growth-inhibitory effects in human leukemia cells [48]. The combination of PPAR*γ* ligand, ciglitazone, and ATRA synergistically reduces the cell growth rates and cell cycle arrest at the G1 phase in HL-60 human leukemia cells, and this is associated with synergistic upregulation of PTEN expression [49]. The combination of 9-cis RA and PPAR*γ* ligand shows significant synergistic effects for the induction of apoptosis in multiple myeloma cells [50]. These reports suggest that the combination of PPAR*γ* ligands plus retinoids holds promise as a novel therapy for some types of hematologic malignancies by activating the transcription of target genes that control apoptosis and differentiation in these malignant cells.

In addition to the hematologic malignancies, a number of preclinical studies indicate the preferable effects by the combination of PPAR ligands plus retinoids on the inhibition of cell growth in solid malignancies, especially in breast caner [52–55]. For instance, Rubin et al. [55] showed that a combination of ligands for PPAR*γ* and RXR inhibits breast aromatase expression induced by tumor-derived factors. Because aromatase activates estrogen biosynthesis, the combination of these ligands may be able to find utility in thetreatment of estrogen-dependent carcinogenesis, such as breast cancer and endometrial cancer [56]. The combination of RXR ligand with ciglitazone also cooperatively inhibits the growth of breast cancer and lung cancer cells by activating the RARE promoter activity and inducing RAR*β*, which plays a critical role in mediating the growth-inhibitory effects of retinoids in various cancer cells [57]. In addition, the synergistic or cooperative effect of RXR and PPAR*γ* agonists for growth inhibition and apoptosis induction is found in colon cancer cells [11, 58]. The detailed effects of PPAR*γ* ligands plus retinoids to inhibit growth in colorectal cancer cells are discussed in the next section.

What are the molecular mechanisms by which the combination of PPAR*γ* ligands and retinoids synergistically induce anticancer effects? Yang et al. [59] reported that the PPAR*γ* and RXR ligands have been shown to differentially recruit subsets of transcriptional coactivators (i.e., p160 by RXR and DRIP205 by PPAR*γ*) to the receptor complex, thus leading to an enhanced transcriptional activation and cellular effects. The transcriptional activity of PPRE is additively induced by treatment with a PPAR*γ* activator plus 9-cis RA, and RXR*α* accumulation, by inhibiting its degradation due to the proteasome system, therefore contributes to the enhancement of PPAR*γ*/RXR activation [60]. The transactivation of the PPRE by PPAR*γ*/RXR heterodimer enhances the expression of the *glutathione S-transferase* gene, which is responsible for the cellular metabolism as well as the detoxification of several xenobiotics and carcinogenic compounds [61]. The findings of these reports suggest that the accumulation of the unphosphorylated form (i.e., functional form) of RXR*α* activates the transcriptional activity of PPRE and thereby enhances the expression of important target genes. The significance of the restoration of RXR*α* by inhibiting its aberrant phosphorylation is reported in the studies using the cell lines of HCC [22, 28], leukemia [29], and colon cancer [11], as discussed below.

## 7. SYNERGY BETWEEN PPAR*γ* LIGANDS AND RETINOIDS IN COLORECTAL CANCER

Among the PPAR targeting therapies, the activation of PPAR*γ* by its ligand is regarded as a potentially useful strategy for the chemoprevention and/or treatment of colorectal cancer because many in vivo and in vitro preclinical studies have demonstrated that PPAR*γ* ligands can inhibit cell growth, cause apoptosis, and thus exert antitumor effects in this malignancy [5, 62–65]. As a result, there has been considerable interest in utilizing the combination of ligands for PPAR*γ* and RXR for the prevention and treatment of colorectal cancer. In fact, it has been reported that in human colon cancer cells the combination of the RXR and PPAR*γ* agonists produces greater efficacy in growth inhibition than either single agent alone, and this is associated with a cooperative reduction in the levels of cyclooxygenase-2 (COX-2) expression and prostaglandin E_2_ (PGE_2_) synthesis [58]. The simultaneous exposure of HT-29 human colon cancer cells to ciglitazone and 9-cis RA results in an increased apoptotic effect and greater inhibition of COX-2 expression, in comparison to cells treated with either ciglitazone or 9-cis RA alone [66]. We recently reported that the combination of 9-cis RA and ciglitazone causes a synergistic inhibition in the growth of human colon cancer Caco2 cells, which express high levels of p-RXR*α* protein, and this is associated with the induction of apoptosis and inhibition in the expression of both COX-2 and c-Jun proteins and mRNAs. The combination of these agents has a synergistic effect in increasing the PPRE activity and decreasing the AP-1 activity. However, we should emphasize that these preferable effects are observed when the phosphorylation of RXR*α* protein is inhibited [11]. Therefore, the inhibition of the phosphorylation of this protein appears to play a critical role in inducing the synergistic growth inhibitory effect in colon cancer cells.

The above findings indicate that the activation of the RXR/PPAR*γ* heterodimer by their specific ligands can decrease the expression of COX-2, which is one of the main mediators in the inflammatory signaling pathway. This seems to be significant because COX-2 plays a critical role in the development of colorectal cancer and might, therefore, be an important molecular target for colorectal cancer prevention and treatment [67]. Recent studies have revealed 2, 4, 6-trinitrobenzene sulfonic acid-induced colitis to be significantly reduced after the administration of both PPAR*γ* and RXR agonists, and this beneficial effect is reflected by the reduction in the NF-*κ*B DNA binding activity in the colon [68]. The inhibition of the *β*-catenin mediated pathway, which promotes the development of colon cancer and is stimulated by COX-2 as well as PGE_2_ [69, 70], by nonsteroidal anti-inflammatory drugs, requires a high-level expression of RXR*α* and PPAR*γ* [71]. Therefore, the activation of the RXR/PPAR*γ* heterodimer by the coadministration of their ligands is clinically useful for the prevention and/or treatment of colon cancer as well as colonic inflammation [72], due to their synergistic effects on the COX-2/PGE_2_ axis.

## 8. SYNERGY BETWEEN PPARs LIGAND AND THE OTHER DRUGS EXCEPT FOR RETINOIDS IN CANCER

In addition to the retinoids, the synergistic effects of PPAR*γ* ligands with other agents have also been reported by many investigators. Girnun et al. [73] found that agonist activation of PPAR*γ* synergistically increases the growth-inhibitory effect of the platinum-based drugs cisplatin and carboplatin in several different types of cancers in both in vivo and in vitro studies. This synergy is associated with the reduction of multiple members of the *metallothionein* gene family expression, which play a role in the resistance of certain cancers to platinum-based drugs [74] by PPAR*γ* [73]. The synergistic or enhancing effects induced by the combination of PPAR ligands plus other conventional chemotherapeutic agents to inhibit cell growth are also reported in several types of cancer cells [75–77]. In addition, it is also reported that the histone deacetylase (HDAC) inhibitors have a synergistic effect with the thiazolidinediones in the activation of PPAR*γ* target genes [78]. In studies using cancer cells, the combination treatment using the PPAR*γ* agonist pioglitazone and the HDAC inhibitor valproic acid has been reported to be more efficient at inhibiting prostate tumor growth than each individual therapy alone [79]. An enhanced growth inhibition is observed when neuroblastoma cells are treated with a PPAR*γ* ligand and a HDAC inhibitor, thus suggesting that a combination therapy to treat neuroblastoma might prove more effective than using either agent alone [80]. These findings suggest that a combination therapy using PPAR*γ* agonists and HDAC inhibitors might therefore be potentially effective for the treatment of some types of human malignancies.

Recently, molecular-targeted therapy is attractive as a new effective strategy to inhibit the growth of cancer cells, and therefore, the combination therapy using such specific molecular-targeting agents plus PPAR ligands may become an important regimen in near future. For instance, the proteasome inhibitor bortezomib, which can inhibit the NF-*κ*B activity, augments the antiproliferative effects of the PPAR*γ* agonist rosiglitazone in human melanoma cells [81]. The dual ligand specific for PPAR*α*/*γ* synergistically enhanced the antiproliferative and proapoptotic effect of imatinib, a specific inhibitor of BCR-ABL tyrosine kinase, in Philadelphia chromosome-positive lymphocytic leukemia and chronic myelogenous leukemia blast crisis cell lines [82, 83]. The growth inhibitory effects by gefitinib, an EGFR tyrosine kinase inhibitor, on the human lung cancer cell line are potentiated by the treatment with PPAR*γ* ligand, and this is associated with an increase in the expression of PTEN, but a reduction in the expression of p-Akt [84]. It is interesting to note that the activation of PPAR*γ* by its ligand causes a dramatic inhibition of the tyrosine phosphorylation of HER2 and HER3 receptors, the other member for the EGFR family of receptor tyrosine kinases (RTKs), in human breast cancer cells [85]. The PPAR*γ* ligand also blocks phosphorylation of other member of RTKs, such as IGF-1R, thereby suppressing the proliferation of breast cancer cell lines [85]. These findings may explain the mechanisms in regard to precisely how the PPAR*γ* ligands can enhance the effects of specific RTK inhibitors, although some other molecules may also play a role.

## 9. IS PPAR PHOSPHORYLATION ASSOCIATED WITH CANCER?

As mentioned above, a malfunction of RXR*α* due to phosphorylation is associated with cancer cell growth and retinoid resistance [11, 22, 27, 29]. However, a question which arises here is whether the phosphorylation of PPARs also plays a role in carcinogenesis and/or resistance to the PPAR ligands. Recent studies have shown that PPARs are phosphoproteins, and their transcriptional activity is affected by several kinases, including ERK/MAPK, both in a ligand-dependent and/or -independent manner [86]. Although the significance of the PPARs phosphorylation in cancer has not been clarified, at least in PPAR*γ*, the transcriptional activity of this receptor is inhibited by phosphorylation [87–89]. Extracellular signals that activate intracellular phosphorylation pathways can influence the degradation process of PPAR*γ* [89, 90]. These reports may suggest that as well as RXRs [22, 27], the phosphorylation-mediated inhibition of transcriptional activity of PPARs is associated with cancer [20]. Hedvat et al. [91] conducted an interesting study, reporting that the activation of PPAR*γ* is sustained by the presence of HER-kinase inhibitor, suggesting that HER-kinase and its downstream ERK/MAPK pathway phosphorylate PPAR*γ* and, therefore, abrogate the effects of PPAR*γ* activity through degradation of this nuclear receptor. In this study, the inhibition of HER-kinase activity was sufficient to inhibit PPAR*γ* protein degradation [91]. These findings suggest that, in future studies, the combination of PPAR*γ*/RXR ligands plus a specific agent which targets the RTKs and/or Ras/MAPK signaling pathway may therefore become a promising strategy to inhibit the growth of cancer cells by inhibiting the phosphorylation of PPARs/RXRs.

## 10. CONCLUSION

**Figure 3:**
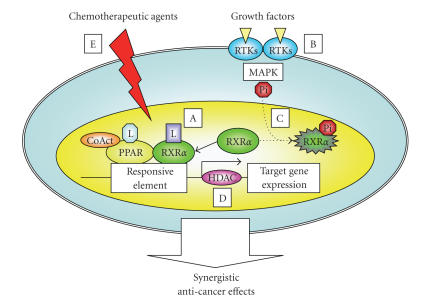
A hypothetical schematic representation of the synergistic anticancer effects of the combination of PPAR ligands plus other agents. When PPARs are activated by ligand binding, they are able to heterodimerize with RXR and activate the target gene expression by binding to the PPRE element. Therefore, the retinoids which bind to RXR may be the most preferable partner for the PPAR agonists (A). However, in some types of cancers, the MAPK pathway phosphorylates RXR*α*, and the accumulated nonfunctional p-RXR*α* interferes with the function of the remaining normal RXR*α*, thereby promoting the growth of cancer cells. The activation of RTKs by their specific ligands (growth factors) can play a critical role in the stimulation of the MAPK pathway. Therefore, the agents which target the activation of RTKs (B) and/or the MAPK pathway (C) restore the function of RXR*α* as a master regulator of nuclear receptors in cancer cells and this will support the synergistic growth inhibition by PPAR and RXR ligands in cancer cells. The HDACs enforce a tight chromatin structure and thereby repress the transcription of target genes controlled by PPAR/RXR. Therefore, the combination of a PPAR agonist plus an HDAC inhibitor is more efficient to inhibit the growth of cancer cells (D). Finally, the conventional chemotherapeutic agents also cause synergistic or enhancing effects to inhibit cancer cell growth by the combination of PPAR ligands (E). L: ligand.

The combined use of two or more agents is often advantageous since it may permit to lower the clinical dosages, thereby decreasing the overall toxicity, and thus providing the potential for synergistic effects between agents. In this review, we made an attempt to show the synergism between PPAR*γ* agonists and other agents (see Figure 3). Among such preferable candidates, retinoids seem to be the best partner of PPAR*γ* ligands in order to exert synergistic antitumor effects. However, phosphorylation of RXR*α* represses the PPAR*γ*/RXR-dependent anticancer effects. In some cases, the inhibition of PPAR phosphorylation may also support the antitumor function of these nuclear receptors. In addition, we should keep in mind the fact that the encouraging results obtained from the combined use of PPAR*γ* agonists plus other agents have been exclusively reported in preclinical studies, and PPAR agonist monotherapy did not achieve a significant result for advanced malignancies in clinical trials, except for a small trial on liposarcomas [7–10]. Therefore, it might be expected that some combination with other agents may lead to breakthrough in the clinical application of PPAR*γ* agonists for chemoprevention and/or treatment of malignancies.

In conclusion, the combination treatment using the PPAR*γ* agonists and other agents might be an effective and promising strategy for chemoprevention and/or treatment of various types of cancers. Future studies will be necessary to improve the anticancer efficacy of PPAR*γ* agonist plus retinoids by combining with appropriate specific kinase inhibitors.

## ABBREVIATIONS

PPAR: Peroxisome proliferator-activated receptor
PPRE: Peroxisome proliferator responsive element
RXR: Retinoid X receptor
RAR: Retinoic acid receptor
p-RXR*α*: Phosphorylated RXR*α*
RXRE: Retinoid X receptor responsive element
RARE: Retinoic acid receptor responsive element
DBD: DNA-binding domain
LBD: Ligand-binding domain
VDR: Vitamin D_3_ receptor
RA: Retinoic acid
ACR: Acyclic retinoid
ATRA: All-trans retinoic acid
MAPK: Mitogen-activated protein kinase
ERK: Extracellular signal-related kinase
JNK: c-Jun N-terminal kinase
HCC: Hepatocellular carcinoma
COX-2: Cyclooxygenase-2
Akt: Protein kinase B
AP-1: Activator protein-1
NF-*κ*B: Nuclear factor-*κ*B
PGE_2_: Prostaglandin E_2_
RTK: Receptor tyrosine kinase
HDAC: Histone deacetylase
HER: Human epidermal growth factor receptor.
